# Development of a phenotype ontology for autism spectrum disorder by natural language processing on electronic health records

**DOI:** 10.1186/s11689-022-09442-0

**Published:** 2022-05-23

**Authors:** Mengge Zhao, James Havrilla, Jacqueline Peng, Madison Drye, Maddie Fecher, Whitney Guthrie, Birkan Tunc, Robert Schultz, Kai Wang, Yunyun Zhou

**Affiliations:** 1 Raymond G. Perelman Center for Cellular and Molecular Therapeutics, Children’s Hospital of Philadelphia, Philadelphia, PA 19104 USA; 2 School of Engineering and Applied Science, University of Pennsylvania, Philadelphia, PA 19104 USA; 3 Center for Autism Research, Children’s Hospital of Philadelphia, Philadelphia, PA 19104 USA; 4 Departments of Pediatrics and Psychiatry, Perelman School of Medicine, University of Pennsylvania, Philadelphia, PA 19104 USA; 5 Department of Pathology and Laboratory Medicine, University of Pennsylvania, Philadelphia, PA 19104 USA

**Keywords:** Autism, Autism spectrum disorder, Natural language processing, Terminology set, Phenotype ontology, Electronic health record

## Abstract

**Abstract:**

**Background:**

Autism spectrum disorder (ASD) is a complex neurodevelopmental condition characterized by restricted, repetitive behavior, and impaired social communication and interactions. However, significant challenges remain in diagnosing and subtyping ASD due in part to the lack of a validated, standardized vocabulary to characterize clinical phenotypic presentation of ASD. Although the human phenotype ontology (HPO) plays an important role in delineating nuanced phenotypes for rare genetic diseases, it is inadequate to capture characteristic of behavioral and psychiatric phenotypes for individuals with ASD. There is a clear need, therefore, for a well-established phenotype terminology set that can assist in characterization of ASD phenotypes from patients’ clinical narratives.

**Methods:**

To address this challenge, we used natural language processing (NLP) techniques to identify and curate ASD phenotypic terms from high-quality unstructured clinical notes in the electronic health record (EHR) on 8499 individuals with ASD, 8177 individuals with non-ASD psychiatric disorders, and 8482 individuals without a documented psychiatric disorder. We further performed dimensional reduction clustering analysis to subgroup individuals with ASD, using nonnegative matrix factorization method.

**Results:**

Through a note-processing pipeline that includes several steps of state-of-the-art NLP approaches, we identified 3336 ASD terms linking to 1943 unique medical concepts, which represents among the largest ASD terminology set to date. The extracted ASD terms were further organized in a formal ontology structure similar to the HPO. Clustering analysis showed that these terms could be used in a diagnostic pipeline to differentiate individuals with ASD from individuals with other psychiatric disorders.

**Conclusion:**

Our ASD phenotype ontology can assist clinicians and researchers in characterizing individuals with ASD, facilitating automated diagnosis, and subtyping individuals with ASD to facilitate personalized therapeutic decision-making.

**Supplementary Information:**

The online version contains supplementary material available at 10.1186/s11689-022-09442-0.

## Background

Autism spectrum disorder (ASD) is a complex neurodevelopmental disorder that affects 1 in 59 children in the USA [1]. Diagnosing and characterizing ASD can be very difficult, as individuals with ASD have markedly heterogeneous presentations of its core symptom domains, i.e., social and communication difficulties and restricted interests/repetitive behaviors [2]. Standardized diagnostic manuals such as the *Diagnostic and Statistical Manual of Mental Disorders*, fifth edition (DSM-5), and the *International Classification of Diseases*, 11th edition (ICD-11), are the gold standards for diagnostic decision-making for ASD and all other mental health conditions. Despite standardized diagnostic criteria, diagnostic decision-making often has modest or low reliability using DSM-5. The field lacks validated biomarkers and other reliable quantitative trait measurements, and thus, diagnosis is based on behavioral observations [3]. As a result, these clinical diagnoses may have low reproducibility among different clinicians assessing the phenotypic characterization in individuals suspected to have ASD [4].

One way to improve the reliability among clinicians is to derive standardized “common language” from diagnostic assessments and clinical notes in the electronic health records (EHR). The standardized language to characterize individuals with ASD could be used in an automated fashion to produce clinical characterization and binary diagnostic decision efficiently, on individual clinical notes, or in batch across large sample of all patients in the EHR meeting minimal information thresholds across a variety of filter. To date, this is a largely unexplored area. Production of such an automated pipeline could be enriched with statistical modeling designed to better describe how similar or different individuals are to one other in a multidimensional space. For example, clustering and related approaches could use quantitative metrices based on a standardized terminology to subtyping individuals with ASD based on their phenotypic characteristics recorded in clinical notes [5–7].

Natural language processing (NLP) technique can assist to create such a standardized vocabulary to overcome time-consuming, inconsistent decision-making barriers among clinicians and communication barriers among different healthcare providers. Although standardized vocabulary is useful in describing the characteristics and phenotypic traits of human medical and mental health conditions, to date, there are no high-quality, standardized terminology sets that can assist in implementing well-defined, comprehensive, and interoperable studies of ASD at a single medical site or across multiple clinical sites. While some terminology sets have been proposed based on experts’ curation — for example, *Barbaresi’s list* used by the NIH Electronic Medical Records and Genomics Network (eMERGE), they are resource-intensive to evaluate each patient by manual evaluation on their EHR data. Moreover, there are license restrictions on using these terminology sets [8].

While some ASD terminology sets have been developed from computational approaches, their limited number of terms is inadequate for more than crude description of ASD phenotypic features. For example, Lingren et al. [9] extracted 831 terms from 302 EHR clinical notes using a clinical NLP tool, cTAKES [10]. The identified ASD terms were also limited by high false-positive rate. Later, to improve the quality of ASD terms, Leroy et al. developed a rule-based NLP approach based on pattern matching from 12 DSM-IV-TR criteria [1]. Their ruled-based approach, however, has limitations compared to machine learning model-based approach, since they cannot recognize novel ASD terms if certain string patterns or lexicons are not part of predefined rule sets in advance. Since existing vocabularies cannot meet the emerging needs of capturing complex phenotypic traits in ASD, it is important to develop a comprehensive, high-quality terminology set to characterize ASD. This problem is not yet resolved, as only a fraction of ASD-relevant clinical descriptions (i.e., phenotype terms) are identified in currently available vocabularies. This impedes the automated diagnosis of ASD, subtyping of ASD, and multidimensional characterization of ASD using digital phenotyping.

In this study, we created a high-quality (HQ) ASD phenotype terminology set from the EHR data of 8499 individuals diagnosed with ASD and evaluated its performance using two separate control cohorts of similar sizes from the Children’s Hospital of Philadelphia (CHOP). In collaboration with a team with significance expertise in the diagnosis and characterization of ASD and related mental health conditions, we performed rigorous data quality control and advanced NLP analysis, using both rule-based and model-based approaches. Through computational validation and manual confirmation by clinicians, we identified 3336 standardized ASD terms, which can be mapped to 1943 unique medical concepts in the Unified Medical Language System (UMLS) [11].

To the best of our knowledge, this is currently the most comprehensive ASD phenotype terminology set extracted from raw clinical notes and the largest ASD patient cohort used to do so. Additionally, we provide this terminology set without license restrictions, so that anybody studying ASD can benefit from the use of standardized terminologies and ontologies, thus enabling cross-institutional studies. The extracted ASD terms were further organized in a formal ontology structure, much like the Human Phenotype Ontology (HPO) [12]. Finally, we demonstrated the utility of our terminology set in differentiating ASD patients from other non-ASD patients with other psychiatric conditions and in mapping individual ASD patients’ phenotype abnormalities to each DSM-5 criterion based on their unstructured clinical notes.

## Methods

### High-quality (HQ) patient cohort selection and clinical notes quality control (QC)

We queried patients’ EHR data from the Epic Clarity database of the Children’s Hospital of Philadelphia (CHOP). Considering that ASD and non-ASD psychiatric patients may have similar psychiatric phenotypes, we queried two types of cohorts: one psychiatric (non-ASD) cohort and one nonpsychiatric cohort, as the controls. Each cohort was identified using the codes of International Classification of Diseases (both ICD-9 and ICD-10-CM). This study was approved by the institutional review board of the CHOP.

We collected all the patients’ clinical notes in the three cohorts: ASD, psychiatric (non-ASD) cohort, and nonpsychiatric cohort. ASD patients may have different types of clinical notes from different departments of the hospital due to different reasons, so we only focused on the most HQ psychiatric notes in the three cohorts. Then, we further excluded nonpsychiatric description sessions in the clinical notes, such as lab test results and treatment plans, keeping only sessions containing psychiatric assessments.

Since most of the raw notes are quite long and are largely free text, we performed rigorous QC for these notes as follows: (1) remove notes with short descriptions (< 100 words), (2) remove the patients with less than 10 visits, (3) remove duplicated descriptions to keep one version, (4) make sure the date of clinical notes match with the date of given ICD code for ASD. We applied the same process on selecting a similar amount of HQ psychiatric notes from the two control groups, as patients in control groups may also have clinical notes from psychiatric department. Since the ratio of male and female in ASD is almost 4:1 and the ASD diagnostic age ranging between 18 months and 18 years old, we selected similar ratio of male and female patients with similar age distribution range for the two control groups. These notes were further evaluated by ASD pediatric clinicians who applied medical protocols to confirm the ASD case status.

### Named entity recognition (NER) and semantic filtering for ASD terms

The task of named entity recognition (NER), for our use case, aims to recognize the phenotypic terms in the psychiatric assessment sections of the EHR clinical notes. From a previous study, we concluded that CLAMP (clinical language annotation, modeling, and processing) [13] is an accurate tool for extracting ASD NEs [14] and renders less false-positive NE terms compared to the other NLP tools, such as cTAKES [10]. Here, we followed the same pipeline as the previous to extract potential ASD NEs from the selected HQ clinical texts. CLAMP maps detected NE terms to the concept unique identifiers (CUI) of UMLS vocabularies. We skipped the NEs that were not mapped to UMLS CUIs. In addition, our study [14] concluded that ASD terms belong to certain semantic types of UMLS. Thus, we selected the CUIs under those semantic types for the downstream analysis (Table 1).

**Table 1:** Selected UMLS semantic types

Abbreviation	Type unique identifier (TUI)	Full semantic type name
acty	T052	Activity
dora	T056	Daily or recreational activity
dsyn	T047	Disease or syndrome
fndg	T033	Finding
hlca	T058	Healthcare activity
inbe	T055	Individual behavior
menp	T041	Mental process
mobd	T048	Mental or behavioral dysfunction
podg	T101	Patient or disabled group
qlco	T080	Qualitative concept
socb	T054	Social behavior
sosy	T184	Sign or symptom

### Statistical analysis for case vs control groups comparison for initial filtering

We calculated odds ratios for each ASD phenotype term for case/control group comparison. The first odds ratio was calculated on the ASD cohort against the nonpsychiatric cohort; the other one was done on the ASD cohorts versus the psychiatric (non-ASD) cohort.

$$Odds\_ Ratio{(NER)}_{C1\ against\ C2}=\frac{freq{(NER)}_{C1}\times \left(1- freq{(NER)}_{C2}\right)}{freq{(NER)}_{C2}\times \Big(1- freq\left({NER}_{C1}\right)\Big)},$$

where *NER* is one concept,

$$C1=\left\{ ASD\ cohort\right\},$$

$$C2=\left\{ Psychiatric\ \left( non- ASD\right)\ cohort\right\},$$

$$freq{(NER)}_{cohort}=\# of\ patients\ of\ which\ NER\ is\ present\ in\ the\ texts\# of\ the\ cohort.$$

We selected the NEs to be the potential list of our ASD terms, if they reached the odds ratio cutoffs: *Odds* _ *Ratio*(*NER*)_*ASD cohort against Non* − *psychiatric cohort*_ > 1.5.

Through pairwise odds ratio comparison on the ASD cohort against the nonpsychiatric cohort and psychiatric (non-ASD) cohort, we obtained an initial list of ASD phenotype terms.

### NLP approach to verify gold standard ASD terms

To obtain a gold standard ASD term set, we collected ASD vocabularies from previous studies [15, 16]. Following DSM-5 criteria, our ASD clinicians used rule-based method to manually examine them and pick the true positive ones for our gold standard ASD term set. DSM-5 guide for ASD diagnosis covers two types of symptoms: social communications and interactions (see criteria A); repetitive, restricted, and ritualized behaviors (see criteria B), and the comorbidity symptoms (see criteria E). Criteria C and D emphasize the time of appearance and the severity of the symptoms in criteria A and B, so we regard criteria C and D as merely complementary to criteria A and B. We truncated descriptive sentence of each DSM-5 criterion into small segments and then extracted initial ASD descriptive terms using CLAMP NER pipeline. Through collaboration with ASD clinicians and using the reference rules in Leroy et al. [1], we further validated ASD lexicon patterns for DSM-5 descriptions.

Next we used deep learning-based BioBERT’s [17] NER algorithm to identify novel NEs that are similar to our gold standard ASD terms from the HQ clinical texts of the ASD cohort. Using this new algorithm is also a way to validate the terms extracted by CLAMP, which will provide an unbiased insight of information extraction. Before performing the task, we randomly labeled 70% of the HQ clinical texts as the training set, 15% of them as the testing set, and 15% as the validation set. We then tokenized the three sets with spaCy (version 2.2.1) and used inside-outside-beginning (IBO) tagging to label the tokens using the gold standard ASD terms. After the task was finished, we de-tokenized the tokens in the testing set, took out the predicted NEs from them, and manually verified them as the true aliases of our gold standard ASD term set. Since the verified aliases came from only 15% of the HQ clinical texts in one such task, we performed this task 10 times as the cross validation to ensure the aliases came from as many HQ clinical texts as possible.

Finally, we used Sent2Vec [18] to convert ASD NEs into embedding vectors, numerical semantic representations of each term. Then we calculated the cosine similarity between the embedding vectors. For each psychiatric phenotype term from our statistical analysis, we calculated such cosine similarities between it and the combined list of gold standard and BioBERT terms one by one. If one of these similarity values was larger than 0.5, we determined the psychiatric phenotype term to be an ASD phenotype term.

### Clustering patient cohorts using our ASD term set by dimensionality reduction method

We assume that an ASD term should be less informative in describing an ASD symptom if it shows up more frequently across all three cohorts. Under this assumption, we chose the term frequency-inverse document frequency (tf-idf) algorithm to analyze our ASD term set:

$${w}_{i,j}={tf}_{i,j}\times {\log}\left(\frac{N}{df_i}\right),$$

where *w*_*i*, *j*_ is the weight for ASD term *i* in the HQ clinical texts of patient *j*, *tf*_*i*, *j*_ is the number of occurrences of the ASD term *i* in the HQ clinical texts of patient j, *df*_*i*_ is the number of patients of whose HQ clinical texts contain the ASD term *i*, and *N* is the total number of patients. This method normalized all the ASD terms across all the HQ clinical texts and gave less weight to those more frequent and higher weights to the rare terms. By applying tf-idf, it produces a *m* × *n* matrix, where *m* is the number of selected patients and *n* is the number of the terms in our ASD term set.

Next we used a nonnegative matrix factorization (NMF) algorithm to split the *m* × *n* matrix into an *m* × *a* matrix and an *a* × *n* matrix, where *a* is the number of reduced dimensions. Then, we plotted the *m* × *a* matrix using the t-SNE method and divided the ASD patients into clusters in the resulting plot.

### ASD phenotype ontology construction

An ontology is a structured term set in which each term has its own properties and relations to other terms. An ASD phenotype ontology can be convenient for computers to understand the term set and facilitate analyzing and diagnosing a subject as having ASD. Thus, we constructed an ASD phenotype ontology using our ASD term set.

Our ASD ontological representation and relationships were generated following the principles of the Open Biological/Biomedical Ontology (OBO) Foundry model (obofoundry.org). ASD vocabularies were organized in a hierarchical structure to display the relationship of broader domains (parent terms) to more granular terms (child terms). Many-to-many relations of different layers of terminology were linked together using the Web Ontology Language (OWL2). The information of an entire ontology can be stored as an RDF or XML file, and we used Owlready2, a Python package, to generate a file that covers the entire information of the ASD ontology. In addition, we visualized the ASD phenotype ontology using Protégé [19], an open-source software for ontology visualization.

## Results

### Workflow and results summary

**Fig. 1:**
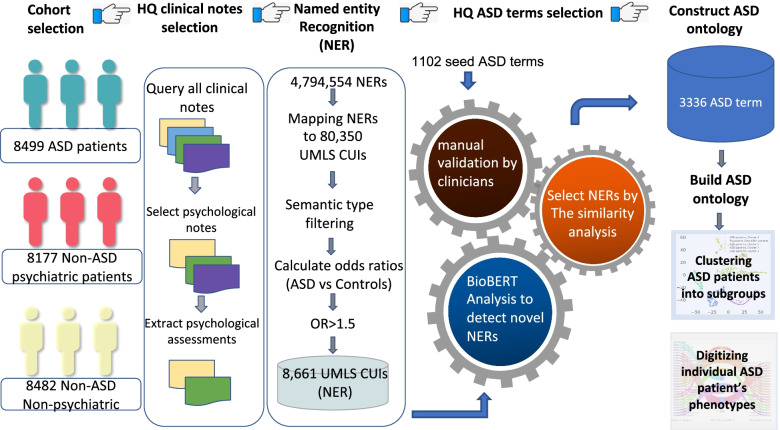
Workflow of ASD phenotype ontology development

The workflow of identifying ASD phenotype terms from HQ clinical text is shown in Fig. 1. Our analysis includes four steps: (1) patient cohort selection, (2) data quality control on the raw clinical notes, (3) identifying ASD phenotype terms by NLP and statistical analysis, and (4) quantifying individual ASD patients’ phenotypes using our terminology set and performing dimensional reduction for patient clustering analysis. After performing cohort selection and rigorous quality control, 8499 ASD patients with 56,958 HQ clinical notes from the psychiatric department were selected. The final ASD terminology set contained 3336 phenotype terms which were further organized into a 5-layer ontology structure based on DSM-5 criteria and our collaborating clinicians’ design. The subsequent analysis showed our ASD phenotype terminology set is better than an existing published ASD vocabulary developed by Lingren et al. [9] in distinguishing ASD patients from non-ASD psychiatric patients. Furthermore, we demonstrated that our terminology set could be used to cluster ASD patients into subgroups and quantitatively map an individual ASD patient’s phenotypic characteristics to DSM-5 criteria.

### Patients and clinical notes availability summary

**Fig. 2:**
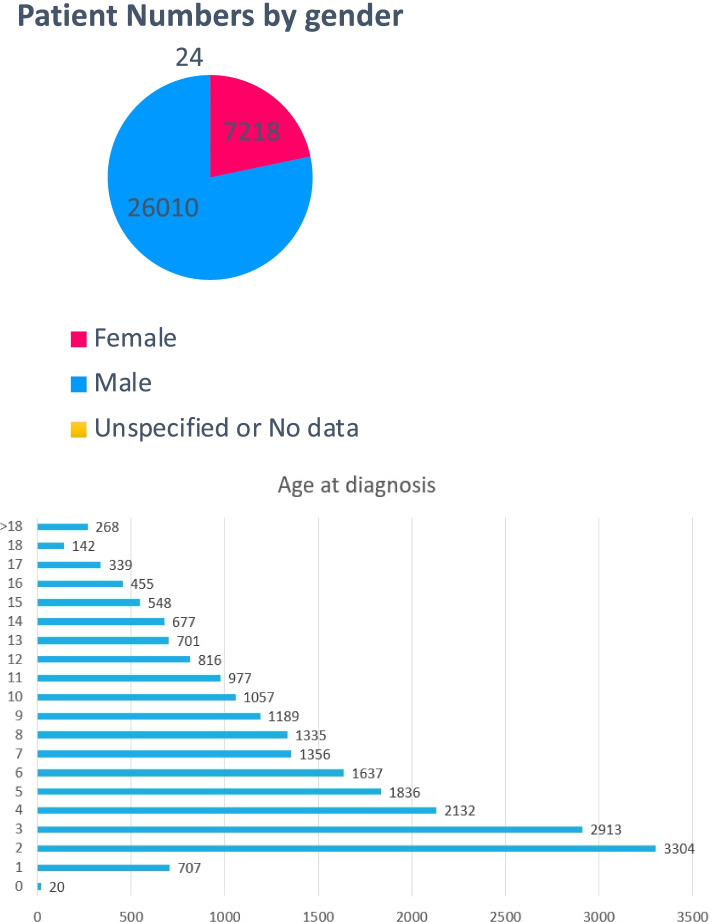
Gender and age distribution in ASD patient cohort. Some patients were diagnosed at very early age, which may represent an artifact of retrospective assignment of ICD codes in EHRs

Initially, we identified 33,230 ASD patients with 3,611,649 clinical notes by ICD-9 and ICD-10-CM codes (see “Methods” for details). In the same way, we queried our EHR database to create two comparison cohorts, a non-ASD psychiatric cohort and a nonpsychiatric cohort, enabling statistical comparisons between these groups. The initial number of patients and clinical notes, as well as their ICD-9 and ICD-10-CM codes, is shown in Table S1. The age and gender distribution of individuals with ASD are shown in Fig. 2. Among these patients, 26,020 are males, 7218 are females, and 24 are of unknown gender in the study cohort. The age at diagnosis was calculated from the value of date at diagnosis of minors date at birth. After QC, the final number of patients, number of HQ psychiatric notes, and gender distributions for the ASD and the other two control cohorts are shown in Table S2, respectively. In total, we validated 56,958 HQ clinical notes from 8499 individuals with ASD, 41,753 HQ notes from 8177 individuals with psychiatric (non-ASD), and 21,028 HQ notes from 8482 individuals without any ASD and psychiatric problems. The distribution of number of HQ psychiatric notes for each ASD individuals can be found in Fig. S1.

### Statistical analysis for ASD terms comparison in case and control groups

Following the same NLP protocol in our previously published paper for ASD literature mining [14], we used CLAMP [13] to extract phenotypic named entities from unstructured documents. Initially, CLAMP recognized 4,794,554 biomedical concept named entities (NEs), and these entities can be mapped to 80,350 UMLS CUIs (concept unique identifiers) based on their similarities with the unique biomedical concepts in UMLS database. We removed those NEs that cannot be mapped to any UMLS CUIs. Also, since not all mapped UMLS CUIs are disease phenotypes relevant, we only kept NEs belong to 12 semantic types (e.g., activity, individual behavior, mental process, shown in Table 1), given that most NEs belong to irrelevant UMLS semantic categories such as lab test, body system, and molecular and chemical types.

Then, we further calculated the frequencies for each NE in the three cohorts and performed odds-ratio (OR) analysis for the ASD cohort against the two control cohorts, non-ASD psychiatric cohort, and nonpsychiatric cohort, respectively. As an initial filtering step, since we wanted a relatively large candidate pool, we used a relatively loose selection criterion. We chose 1.5 as the OR cutoff, because our hypothesis is that if the frequency of a NE is 50% higher likelihood in ASD cohort than in non-ASD cohort, we consider that NE is potentially associated with ASD. A list of 8661 NEs passed the initial odds ratio cutoff, *OR* > 1.5. Results showed the NEs referring to ASD communication characteristics such as “nonverbal/nonverbal communications” had the largest odds ratios between ASD vs. nonpsychiatric group comparison. While NEs are describing restricted/repetitive behavior features, such as “autistic behavior,” “stereotyped phrases, repetitive language, have the largest odds ratio from ASD vs. non-ASD psychiatric group comparison. Thus, impairment of social interactions and communication behaviors, as well as stereotyped behaviors, is the most significant and consistent psychiatric symptoms in the EHR of ASD patients compared to non-ASD population. This observation, purely made from clinical notes of EHR, is consistent with the DSM-5 ASD diagnosis criteria.

### NLP embedding analysis to further identify ASD specific terms

Filtering NEs based on the odds ratio value of CLAMP output is not optimal, since the cutoff selection is arbitrary, and it is possible that some false-positive terms were included while some true positive terms were excluded in the initial list of 8661 ASD terms. Because human manual evaluation on all these terms is labor-intensive, we used semiautomated approaches to recognize some novel ASD terms and exclude false-positive ones automatically, based on a limited number of true positive ASD terms labeled by human experts. In collaboration with three clinicians specialized in ASD, we manually examined and verified 1102 ASD terms collected from PubMed literature searching and organized them into four main categories based on types of terms (Table 2).

**Table 2:** Clinician curated classification categories and examples

Classification	Example lexicon			
Terms relating to an ASD diagnosis	Autism, Autistic, PDD-NOS Pervasive development disorder ASD, Asperger, syndrome, phenotype			
Terms related to ASD diagnostic features found in DSM 4/5, such as social communication impairment, restrictive behaviors, etc.	Language		Toy	Stereotype
	Speech		Play	Repetitive
	Verbal		Motor	Resist
	Communicate		Attention	Deficit
	Express		Gaze	Rigid
	Social		Develop	Difficult
	Interact		Self	Aggress
	Behave		Preoccupation	Delay
	Contact		Reciprocal	Obsess
			Ritual	Impair
			Routine	Inappropriate
			Sustain	Poor
			Ability	Inflexible
Terms for behaviors that could be related to or associated with ASD but not found in DSM 4/5	Hand	Neuro	Facial	Toe
	Head	Muscle	Eye	Body
	Face	Sleep	Arm	Finger
Terms related to non-ASD psychological or neurological diagnoses	Epilepsy, stress, emotional, seizures Suicide, anger, ADHD, anxiety Depress, fear, anxious, hyperactive			

Using these 1102 terms as the “seed” list, we further performed two steps of NLP analysis to warrant the delivery of high-quality terms: (1) using BioBERT’s NER [17] to train ASD patients’ clinical notes, we identified and verified 302 novel ASD terms from clinical notes that were not captured in the initial list. These 302 terms were added to the “seed” list, which was considered as the new gold standard list; (2) using the BioSent2Vec embedding model [18], we converted all these ASD terms into 700 high-dimensional vectors and calculated the semantic cosine similarities between the initial 8661 ASD NEs and the new gold standard ASD terms. Among these 8661 NEs, 1943 ASD terms showed high similarity with ASD gold standard terms, so we consider them as the true positive ASD NEs with high confidence.

The final list of 3336 NEs with high confidence is shown in Table S3. The newly recognized ASD phenotype terms have ID starting with “ASD,” while the gold standard terms have IDs starting with “S.” Each ASD NE has statistical information such as the percentage of patients containing this term in each cohort and the odds ratio value for ASD VS. control group comparison. The correlation score for each ASD NEs mapping to the DSM-5 criteria comes from the cosine similarity value of embedding analysis. To obtain the statistical data for the new recognized terms, we run CLAMP on clinical notes again using dictionary look-up function.

### Patient clustering

**Fig. 3:**
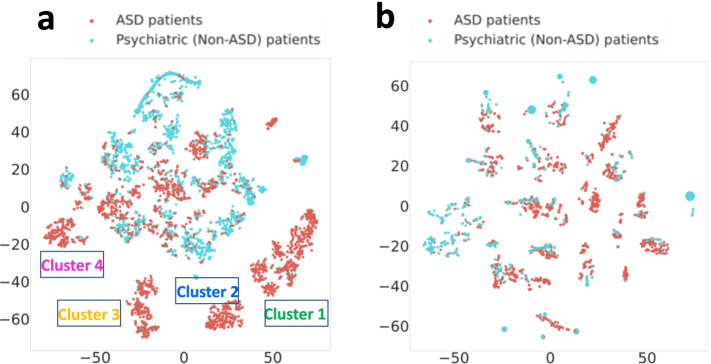
Comparison of t-SNE clustering analysis for top 2000 ASD patients and 2000 psychiatric (non-ASD) patients using our terminology set (**a**) and using Lingren’s terminology set (**b**). Since not all the patients contain the ASD vocabulary developed by Lingren et al., we only analyzed patients containing these terms. Results showed that our terminology set separates ASD patients from general psychiatric (non-ASD) patients much better than Lingren’s list. From the t-SNE plot, we can see ASD patients can be further divided into 4 subgroups; however, one group of ASD patients (cluster 4) is mixed with non-ASD psychiatric patients

To show the utility of our terminology set in characterizing ASD phenotype, we selected 2000 ASD patients and 2000 non-ASD psychiatric patients whose clinical notes contain the most ASD phenotypic concept information. We used CLAMP to extract ASD terms that map to the terminology set from psychiatric notes of each patient. In this case, each patient contains a list of standard ASD terms. A binary data matrix with 4000 rows (patients) and 3336 columns (terms) was generated based on whether a particular term is presented or absented in the patient’s notes. We then used the TF-IDF (term frequency-inverse document frequency) method to transform the data matrix and performed nonnegative matrix factorization (NMF) to cluster patients. Next, we used a t-SNE plot to demonstrate ASD and non-ASD psychiatric patient clusters for data visualization. Figure 3 showed the comparison of patient clusters using our terminology set and Lingren’s ASD list. As shown in Fig. 3a, we can see that ASD patients are mostly clustered in the lower half of the plot, while the non-ASD psychiatric patients are clustered on the upper half. We also observed four distinct subgroups of ASD patients, while a subset of ASD patients is mixed with non-ASD psychiatric patients. However, there are no clear cluster patterns that can be observed using Lingren’s ASD list, as shown in Fig. 3b. Of note, because Lingren’s list contains very limited ASD terms, only a fraction of patients in ASD and non-ASD groups contains features from the Lingren’s list. For example, only 191 ASD terms from Lingren’s list can be found in 1960 ASD group and 999 psychiatric non-ASD group, while in comparison, 1196 ASD terms from our list can be found in both ASD and non-ASD psychiatric groups with 2000 patients. It suggests that our terminology set is more efficient in identifying ASD patients’ phenotypes and therefore has a better separation for ASD patients from general psychiatric patients.

We further examined the subgroups of the 2000 ASD patients and explored how these subgroups of patients map to the DSM-5 guidelines. The relationship between clusters and DSM-5 guidelines is determined by their NE similarities. The descriptive sentence of each DSM-5 criterion was truncated into a list of ASD NEs (Table S4), then these NEs were converted to high-dimensional vectors using the BioSent2Vec embedding approach. To determine the relationships between ASD patients’ phenotypes and DSM-5 guideline, we mapped phenotype terms with the highest cosine similarity value to the criteria. Table S5 displays examples of ASD phenotype terms that are matched to each DSM-5 criterion for ASD.

**Fig. 4:**
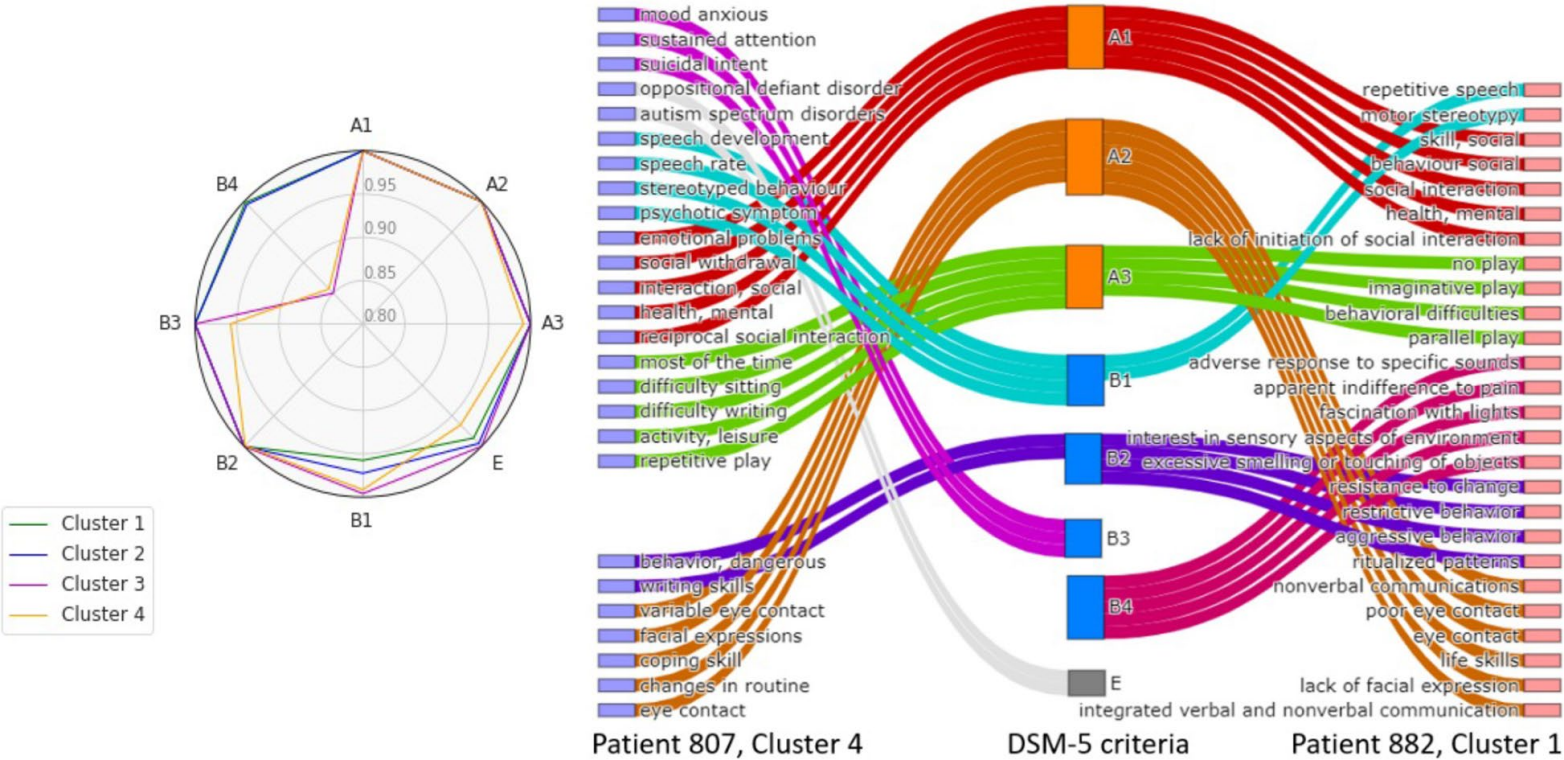
Mapping subgroup of ASD patients to DSM-5 guideline. a The percentage of subgroups of ASD patients in each cluster that maps to DSM-5 individual criteria. b As an illustrative example, we quantified individual patient’s ASD characteristics to DSM-5 guideline for patients in cluster 1 and cluster 4

To show the real-world notes of patients containing key diagnostic information, we generated a radar plot to display the mapping from summarized patients’ phenotypes to DSM-5’s individual guideline. As long as a patient’s notes contain any phenotypic terms that are the children of the parental (root) category “social interaction” in the ontology, we will assign this patient to the DSM-5 A1 criteria “social interaction.” The value in radar plot means the percentage of patients that matches to a certain criterion in DSM-5. As the radar plot is shown in Fig. 4a, nearly all patients from the four clusters had ASD phenotype terms under DSM-5 criterion A1 (social interaction), A2 (social communication), A3 (social relationship), and B2 (ritualized behaviors). Meanwhile, the percentage of patients in each cluster varied in terms of having phenotype terms under DSM-5 criteria B1 (repetitive behaviors), B3 (fascination and preoccupation), and B4 (unusual sensory and comorbidities). This corroborates the ASD diagnosis criteria in DSM-5; subjects should manifest symptoms in all A1, A2, and A3 and two of B1, B2, B3, and B4. Figure 4b shows how individual patients from different subgroups map to DSM-5 criteria based on the phenotype terms extracted from their clinical notes.

### Better ontology structure facilitates ASD phenotype interpretation

**Fig. 5:**
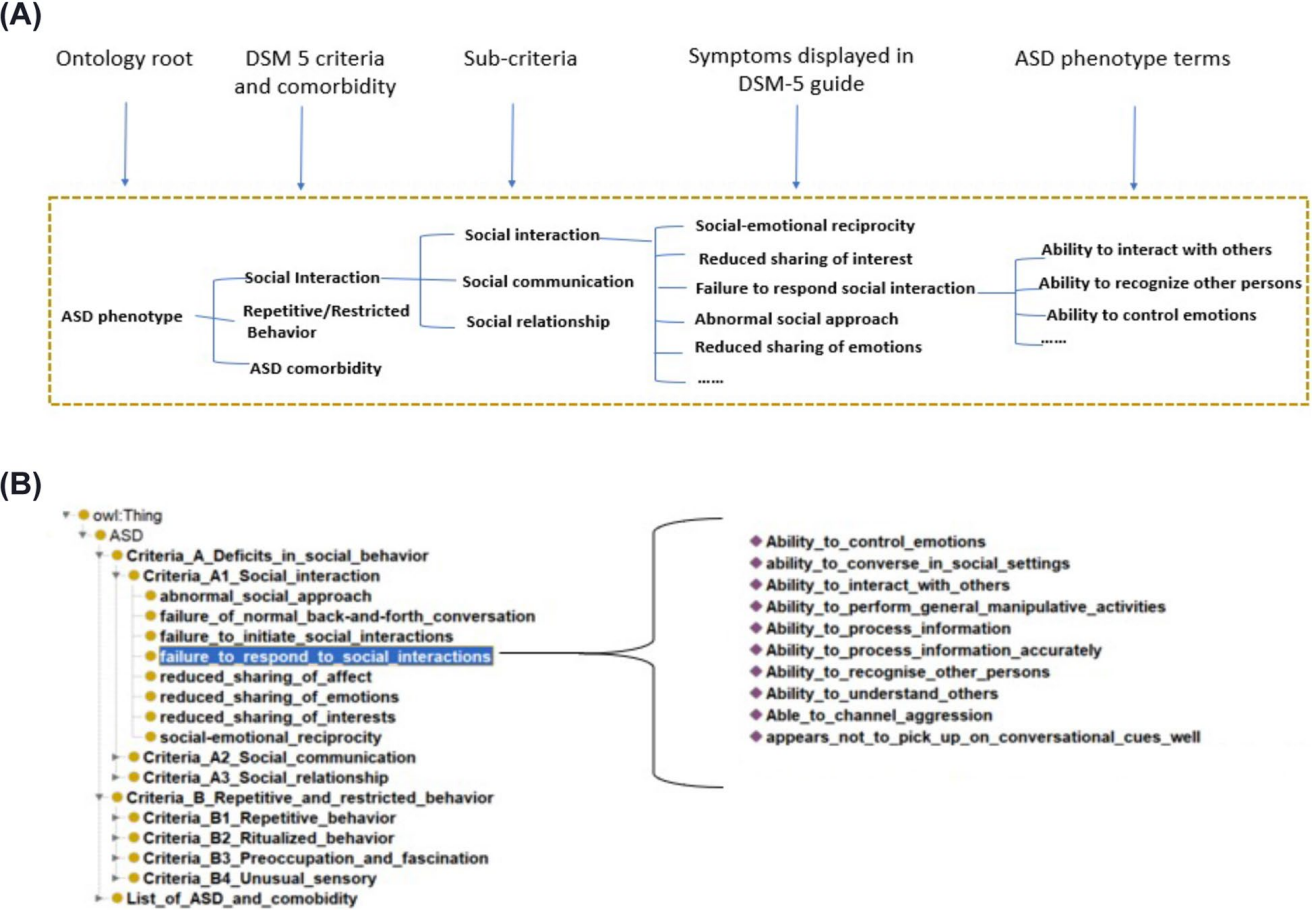
Five levels of ASD phenotype ontology developed in our study. **A** Example of ASD phenotype ontology. **B** Examples of our ASD phenotype ontology displayed in the Protégé software for ontology analysis

The ASD phenotype ontology is a data tree with five levels (Fig. 5A). The first level contains only the root node, “ASD.” The second level consists of 3 nodes: “social interaction” represents criterion A in DSM-5, “repetitive behavior represents criterion B,” and “ASD and comorbidities” refer to the different names of ASD and its comorbidities and represent criterion E. The nodes in level 3 are the sub-criteria in DSM-5 domain for ASD. In level 4, the nodes are ASD phenotype terms extracted from DSM-5 guideline by CLAMP. The nodes in the last level are our ASD phenotype terminology set learned from ASD patients’ clinical notes, which is the most important level as the one that can provide a richer characterization of ASD. Each node has the following properties: the CUI, one of the standard names in UMLS database, the semantic type(s) of CUI, the category of DSM-5 guideline, and the odds ratios of the term as used in ASD vs control EHR notes. The entire information of the ontology is stored in both an RDF and an XML file. These file formats can be imported to Protégé, an open-source ontology editor and a knowledge management system, where the ontology structure can be viewed easily (Fig. 5B).

## Discussion

The current study focused on autism spectrum disorder (ASD), which is a developmental disorder characterized by deficits in both social communication/interaction and restricted/repetitive behaviors, as well as a broad range of associated features, such as motor problems and other psychiatric symptoms and comorbidities. Diagnosis of ASD is based on observations of ASD patients’ characteristics following criteria laid out in the DSM-5. However, the diagnostic evaluation is not always achieving full agreement across clinicians, as might be expected of its subjective nature [2]. It is also time-consuming and reliant on highly trained clinical experts in limited supply, resulting in long wait times for an evaluation. Therefore, the ASD community needs a standardized vocabulary to represent ASD phenotypic trait and to facilitate precision medicine approaches for treatment decision and outcome assessment.

One limitation of currently published ASD terminologies is their inability to comprehensively cover most ASD phenotypic traits [11]. Also, the ASD phenotypic traits described in the DSM-5 criteria are used for illustrative purpose; therefore, only a small amount of ASD terms can be collected from the DSM-5 documents. Many novel ASD trait descriptions, like “difficulty switching between activities,” were not identified as an ASD phenotype term in these previous studies or DSM-5 diagnostic documents but reflecting the problem with “repetitive, restricted and ritualized behaviors” in the DSM-5 criteria B. Due to the lack of large-quantity and high-quality EHR data as the exhaustive example of ASD traits description resources, only a fraction of ASD-relevant phenotype terms is identified in currently available vocabularies. Another consequence of lower-quality vocabulary is that often terms arise that are not actually descriptive of the ASD-specific phenotype but are significantly associated with non-ASD psychiatric phenotypes, such as “aggressive behavior.” This greatly impedes the precision diagnosis of ASD using EHRs. Large-scale EHR data from our in-house ASD patient database made it possible to identify NEs from the patients’ clinical notes.

In this study, we were able to deliver a list of ASD phenotype terms organized in an ontology structure. The ontology structure is helpful for faster and accurate ASD phenotypic information queries. In addition, we used both rule-based and model-based NLP approaches for ASD NEs’ information extraction and prioritization with statistical, evidential support from EHRs, which is an improvement upon existing approaches. Our results showed that our identified ASD terminology set performed better than other terminology sets in separating ASD patients from general non-ASD psychiatric patients from dimensional reduction analysis. Sub-group patterns for ASD population also can be observed and aligned to DSM-5 criteria, which further showed the utility of our terminology set in assisting ASD precise diagnosis. Furthermore, our phenotype terminology set on ASD was organized in an ontology structure, allowing users to query comprehensive ASD phenotypes from more general to more specific descriptions.

Although NLP and machine learning methods displayed their unique advantages in automating disease diagnosis, these approaches still require clinicians in the loop to further refine the machine output. Therefore, we applied the human-machine complementary strategy to assure the success of high-quality ASD vocabulary delivery. Also, to achieve high prediction accuracy of automated diagnosis for ASD, there is still a long path to go. Our results showed some subgroup patterns of ASD patients using our developed terminology set; however, we can still observe that some ASD patients were mixed with the general psychiatric diseases population. Modeling more types of ASD patients’ clinical data such as facial images, behavioral videos, and genomic data, together with clinical notes, might be helpful to improve the diagnostic accuracy in the future. We also planned to validate our ASD terms on an external independent set of clinical notes with IRB approval. Further work would be helpful to involve multicenters ASD data to increase the predictive power, yet data aggregation challenge should be well thought, as EHR documentation and systems and clinic structures differ across healthcare systems. Further improvement could be focusing on the terms’ generalization to different types of notes or a diverse of diagnostic instruments which involves more phenotype features.

In addition, we certainly agree that it would be more accurate for ASD screening when they go through standard diagnostic instrument screening, such as ADI-R, ADOS, and CBC. However, it is too expensive to apply these standard approaches on such a large population cohort to identify potential candidates suitable for these standard diagnostic instruments. Several days and additional costs are often spent on manually collecting diagnostic instruments measurements from each ASD candidate. Therefore, the use of patients’ clinical notes from the EHR database is a promising step towards identifying potential patients for follow-up and for classifying patients based on documented phenotypic presentations. Although data is not shown in this project, we have collected 14 instruments, such as ADI-R, ADOS, CBCL, and VABS-II, which were applied to ~1000 patients from EHR database who also enrolled in our internal research cohort. It is our plan (with IRB approval) to follow the same computational framework and validate these ASD terms on these ~1000 patients from a research cohort. Despite the above limitations, our study represents an essential step for improving diagnosis and digital characterization of ASD to facilitate the implementation of individualized therapeutic or intervention strategies. We hope our approach can overcome the inconsistent decision-making barriers among clinicians and provide a pivotal attempt for ASD automated diagnosis by AI technology.

## Conclusion

In this study, we used NLP techniques to identify and curate ASD phenotypic terms from raw clinical notes in EHR for individuals with ASD. In total, we identified 3336 ASD terms linking to 1943 unique medical concepts, which represents among the largest ASD terminology set to date.

The extracted ASD terminology set was further organized in a formal ontology structure format. We further performed NMF clustering analysis to classify individuals with ASD using ASD phenotype ontology. Results showed that these terms could be used in a diagnostic pipeline to differentiate ASD from other psychiatric disorders. Our ASD phenotype ontology can assist clinicians and researchers in characterizing individuals with ASD, facilitating automated diagnosis, and subtyping individuals with ASD to facilitate personalized therapeutic decision-making.

## Supplementary Information

**Additional file 1: Fig. S1.** Number of notes distribution for the two control cohorts. **Table S1.** Data resources and ICD code. **Table S2.** High quality (HQ) clinical notes selection and gender distribution for the three cohorts.

**Additional file 2: Tables S3-S5.**

## Data Availability

Not applicable.

## Abbreviations

ASD: Autism spectrum disorder
NLP: Natural language processing
EHR: Electronic health record
NER: Named entity recognition
DSM-5: *Diagnostic and Statistical Manual of Mental Disorders*, 5th edition
NMF: Non-negative matrix factorization
