# Supplementary material for: Development of a phenotype ontology for autism spectrum disorder by natural language processing on electronic health records
Source: J Neurodev Disord. 2022 May 23;14:32. doi: 10.1186/s11689-022-09442-0 (PMC9128253; doi:10.1186/s11689-022-09442-0)
Supplement: Supplementary file 1 — Additional file 1: Fig. S1. Number of notes distribution for the two control cohorts. Table S1. Data resources and ICD code. Table S2. High quality (HQ) clinical notes selection and gender distribution for the three cohorts. [file 11689_2022_9442_MOESM1_ESM.docx]

**Supplementary Tables**

**Table S1. Data resources and ICD code**

| **Cohort** | **Patient Number** | **Number of clinical notes** | **ICD-10-CM** | **ICD-9** |
| --- | --- | --- | --- | --- |
| **ASD** | **33,230** | **3,611,649** | **F84*** | **299*** |
| **Other psychiatric diseases, non-ASD** | **188,041** | **33,802,821** | **F01-F99* (excluding F84)** | **290 – 310***  **(excluding 299)** |
| **Non psychiatric diseases, non-ASD** | **737,078** | **32,703,302** | **excluding F** | **excluding 290 - 310*** |

*: The ICD 9/10 codes listed in the table include fractional digits, i.e. F84 includes F84.05.

**Table S2. High quality (HQ) clinical notes selection and gender distribution for the three cohorts.**

| **Cohorts** | **# patients** | **# notes** | **female** | **male** |
| --- | --- | --- | --- | --- |
| ASD | 8499 | 56958 | 1844 | 6655 |
| Psychiatric, non-ASD | 8177 | 41753 | 2111 | 6063 |
| non-Psychiatric, non-ASD | 8482 | 21028 | 4818 | 3656 |

**Figure S1. Number of notes distribution for the two control cohorts.**


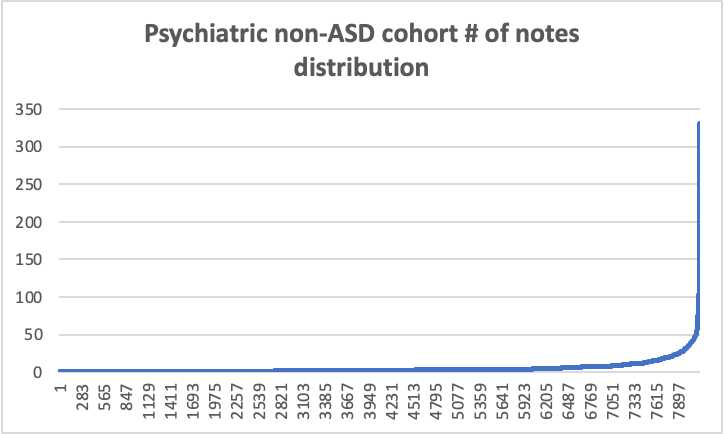

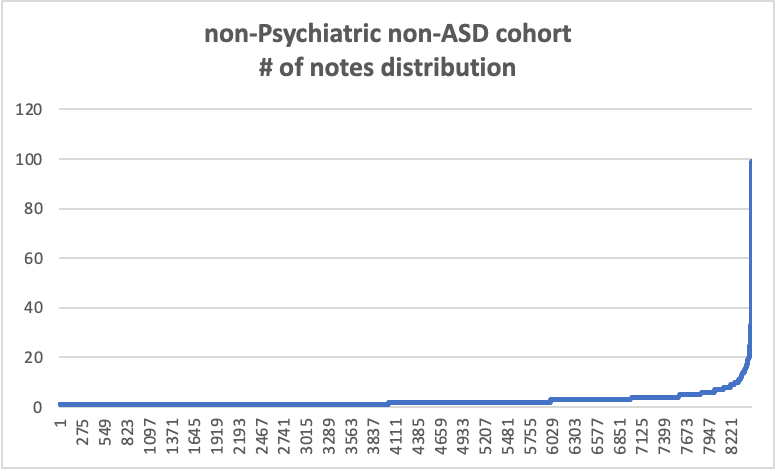


We can see from the figures that most patients have very few HQ clinical notes. However non-Psychiatric non-ASD cohort contains less HQ Psychiatric notes than the Psychiatric non-ASD cohort (proportion test, p-value < 2.2e-16), which is consistent with the reality observations.
